# Postdiction in Visual Awareness and Intrinsic Religiosity

**DOI:** 10.1111/cogs.13171

**Published:** 2022-06-23

**Authors:** Szabolcs Kéri

**Affiliations:** ^1^ Department of Cognitive Science Budapest University of Technology and Economics; ^2^ Hungarian Association for Behavioral Cognitive, and Schema Therapy; ^3^ Nyírő Gyula National Institute of Psychiatry and Addictions; ^4^ Department of Physiology University of Szeged

**Keywords:** Postdiction, Beliefs, Atheism, Religious cognition

## Abstract

The mistiming and fusion of predictive thought and actual perception result in postdiction in awareness, a critical factor in the emergence of nonrational beliefs. Individuals with delusive thinking tend to experience a temporal reversal of prediction (“I guess the rain will fall.”) and real perception (“I feel the rain falling.”), incorrectly showing conviction that their predictions are correct. It is unknown how postdiction is related to religious cognition with a particular reference to intrinsic religiosity when religious beliefs and values are master motives and fundamental frameworks of life. Using a temporal decision‐making task, we investigated a group of religiously committed individuals, atheists, and people from the general community. Results revealed higher postdiction at short thought‐precept time intervals in the intrinsic religious group relative to the atheists. Intrinsic religiosity, but not delusive thinking, was predicted by postdiction in both religious individuals and the general population. These results indicate that people who display pronounced thought‐percept reversal and fusion feel that they are close to a higher power and the sacred.

## 1 Introduction

The seminal question in the cognitive science of religion (CSR) is why religious beliefs, values, rituals, and behaviors are so prevalent in human culture. Some theories indicate that religious beliefs are formed by theleological reasoning, minimal counterintuitiveness of concepts, and a hypersensitive agency detection device (HADD) that postulates intentional agents without physical bodies (spirits, ghosts, and gods) to explain emotionally and existentially salient life events (Barrett, 1999, 2000, 2011; Boyer, 2001, 2003; McCauley & Cohen, 2010). Nevertheless, the fundamental underlying function behind religious and other types of abstract cognition is that humans construct internal pictures and propositions, commonly referred to as beliefs (Spector, 2012; Wittgenstein, 1922). In naturalistic epistemology, beliefs are mental representations of the observable reality that an individual or a group considers true (Churchland & Churchland, 2013; Connors & Halligan, 2014; Sacks & Hirsch, 2008). The perception of the external world is essential in the emergence of such empirical beliefs, which are building blocks of socially, culturally, and historically embedded narratives, a system of language‐bound conceptual beliefs (Seitz & Angel, 2020). Religious beliefs are conceptual representations implementing a framework that promotes meaning‐making and purpose provision for personal and collective experiences (Oviedo & Szocik, 2020; Paloutzian, 2005).

Human belief formation is not entirely rational. Based on extensive work in the cognitive science of belief formation, religion, and delusive thinking (Atran, 2002; Barrett, 2000; Boyer, 2001; Connors & Halligan, 2017; Seitz & Angel, 2015), Shermer (2011) proposed an evolutionary rooted belief engine in the brain that actively seeks structure, predictability, and meaning in environmental information flow. The belief engine forms patterns (patternicity) and attributes events to intentional agents (agencity) even when there are no statistical regularities in the environment and no agents who intentionally cause something. For example, earthquakes, tornadoes, and car accidents are not caused by an invisible power with intentions, thoughts, and feelings (Grayling, 2011; Shermer, 2011). When one has formed a belief, the engine in the brain tends to reinforce it by rationalization and picks up supportive evidence against any contradiction to the truth content of the belief. Shermer (2011) thus claimed that our beliefs determine our reality, a form of belief‐dependent realism. This theory explains why and how people create beliefs of supernatural powers or hidden conspiracies that rule and guide personal, natural, and historical existence by finding regularities in noise and supposing deterministic intentional agents behind probabilistic events. Therefore, the general view is that religious cognition reflects a culture‐driven activation of the brain's evolutionary rooted information processing systems. For example, gods' perceived intentions and involvement in personal life are generated by the Theory of Mind and agent detection systems (HADD), whereas mystical experiences and doctrinal knowledge are housed in cognitive faculties and neural networks for abstract semantics and mental imagery (Kapogiannis et al., 2009; Rim et al., 2019).

As discussed above, the interaction between perception and thought is a cornerstone in the naturalistic epistemology of belief formation. Therefore, mistiming of perception and thought, resulting in postdiction in awareness, may be critical in nonrational beliefs (Bear & Bloom, 2016; Bear, Fortgang, Bronstein, & Cannon, 2017; Cleary, Huebert, McNeely‐White, & Spahr, 2019; Eagleman & Sejnowski, 2000; Grabot, Kayser, & van Wassenhove, 2021; Shimojo, 2014). Bear et al. (2017) provided a succinct description of postdiction bias: “Imagine that, as you leave your house, a few raindrops fall on your skin. You may have the thought that you should go grab your umbrella. Such an observation is completely ordinary and unlikely to encourage any odd beliefs about how the world works. However, a minor alteration to the order in which this perception and thought arise might produce a dramatically different outcome. Mistakenly thinking that you knew to grab your umbrella before you felt raindrops might inspire the belief that you have an exceptional ability to predict the weather or even that you are clairvoyant. More generally, someone who systematically misperceives herself as successfully predicting an event like the weather could come to hold exaggerated or even delusional beliefs about her knowledge or agency.” In other words, people may experience a temporal reversal of prediction (“I guess the rain will fall.”) and perception (“I feel the rain falling.”), called postdiction.

Notably, a simple psychophysical postdiction task provides essential information about how people form higher‐level nonrational beliefs about themselves, others, and the world (Bear et al., 2017). During this task, five empty squares appear on display, and participants predict which one of the five squares will turn red. Interestingly, people overestimate their predictive abilities, especially when the time interval between the appearance of empty squares and the color is short (Bear et al., 2017). The critical assumption is that individuals who tend to confuse their anticipation (an internally emerging feeling about which square will be red) and perceptual experience (an external change of color) would also display nonrational higher‐level beliefs, often at the level of delusions (e.g., magical thinking, alien control, supernatural powers, thought broadcasting, and future telling). Indeed, Bear et al. (2017) showed that individuals with enhanced postdiction (i.e., when people believed that they correctly predicted an event they perceived) scored higher on a scale measuring nonclinical delusional thinking.

A critical issue is whether postdiction is not only relevant to delusions but also to the understanding of religious beliefs (Dawkins, 2006). This question stems from theories suggesting a relationship between delusions and religious beliefs if one conceptualizes them as “a false belief based on incorrect inference about external reality” (American Psychiatric Association, 2013). However, cultural acceptance and social adaptation that mediate bonding, alliances, and cooperation within a group are fundamental distinguishing features between delusions and religious beliefs: delusions are socially maladaptive and “not ordinarily accepted by other members of the person's culture or subculture.” (American Psychiatric Association, 2013) Although the American Psychiatric Association's distinction is apparent, evidence from clinical psychology, cultural anthropology, and cognitive science indicate a continuum between healthy religious thoughts and psychopathological phenomena. The boundary between normal and abnormal is often blurred, and sometimes biased valuation, preoccupation, and distress, and not the core belief content, define delusion (e.g., some people with paranoia can be persecuted) (Connors & Halligan, 2017; McCauley & Graham, 2020; McKay & Ross, 2021).

Intrinsic religiosity is particularly relevant when considering the relationship between religious cognition and delusive thinking. People with high intrinsic religiosity consider religious thoughts, values, and feelings toward the sacred as a fundamental framework, organizing principle, and meaning‐making factor in their life (Allport & Ross, 1967; Gorsuch, 1994). Although there is a link between mental health, conscientiousness, agreeableness, self‐control, and intrinsic religiosity (McCullough & Willoughby, 2009), delusive thinking may accompany some extreme manifestations of intrinsic religiosity. For example, if one scores high on a scale item stating “In my life, I experience the presence of the Divine (i.e., God)” (Koenig & Büssing, 2010), it can be a sign of adaptive spirituality, but also may mark extreme preoccupations, distress, and detachment from reality.

The rationale of our study was to investigate the relationship between postdiction and intrinsic religiosity. In the first experiment, we investigated postdiction and delusive thinking in people with extreme intrinsic religiosity and compared them to highly religious people with less intensive intrinsic religiosity and atheists. In the second experiment, we recruited a larger sample from the general population to replicate the findings of the first experiment. The main hypothesis was that there is a positive link between postdiction and intrinsic religiosity (i.e., people with high intrinsic religiosity show greater postdiction in visual awareness).

## 2 Methods

### 2.1 Participants

There were two main groups of volunteers: (1) highly religious and atheist individuals matched for demographic measures, and (2) people from the general population. In the first group, we enrolled 100 individuals who declared themselves deeply religious from Hungary's Roman Catholic, Protestant, and Pentecostal communities using local networks and pastoral care services. Fifty individuals were characterized by utmost intrinsic religiosity and 50 volunteers with less intrinsic religiosity. We used the modified Duke University Religiosity Index (DUREL) to define intrinsic religiosity, which taps on organized religious activity, individual religious activity, and intrinsic religiosity. People with utmost intrinsic religiosity achieved the highest possible scores on each intrinsic religiosity item of the DUREL (“experience of the Divine, religious beliefs behind the whole approach to life, and carrying religion over into all other dealings in life”) (Koenig & Büssing, 2010). We also included 50 participants who stated that they were atheists. Atheists achieved the lowest possible scores on each DUREL item (Table 1).

**Table 1:** Characteristics of the participants

	Nonintrinsic religious (*n*=50)	Intrinsic religious (*n*=50)	Atheist (*n*=50)	General population (*n*=350)
Age (years)	39.3 (13.4)	41.1 (14.0)	39.0 (12.6)	36.2 (14.5)
Education (years)	12.3 (3.4)	12.2 (3.1)	12.7 (3.8)	13.1 (3.7)
Beck Depression Inventory‐II	9.3 (3.9)	9.0 (4.2)	8.5 (3.7)	9.9 (4.7)
Beck Anxiety Inventory	4.1 (2.3)	3.5 (1.8)	3.7 (2.2)	5.4 (2.9)
Peters et al. Delusion Inventory	51.7 (34.1)	56.1 (37.9)	57.6 (33.4)	55.6 (34.3)
Working memory index	108.5 (13.2)	108.2 (12.2)	109.8 (11.7)	101.7 (10.6)
DUREL organized religious activity	3.6 (1.7)	3.9 (1.8)	0^b^	3.1 (1.5)
DUREL nonorganized religious activity	4.2 (1.8)	4.7 (1.7)	0^b^	3.5 (1.5)
DUREL intrinsic religiosity	2.9 (1.0)	5.0 (0.0)^a^	0^b^	3.0 (1.4)

*Note*: Data are mean (standard deviation). DUREL––Duke University Religion Index. The groups did not differ in age, education, working memory index, depression, anxiety, and delusive thinking (*p*s > .2).

^a^ In the intrinsic religious group, the DUREL intrinsic religion scores were the maximum (5 points) in each participant (*SD* = 0.0). The remaining DUREL scores did not differ between the intrinsic and nonintrinsic religious group (*p*s > .2).

^b^ In the atheist group, the DUREL scores were zero in each participant (*SD* = 0.0).

The second group was recruited from the general population. We used digital social media advertisement, and random digit dialing to obtain a representative sample for age, gender, education, income, rural and urban geography, and perceived health (all Cramer V‐values < 0.1). The DUREL scores indicated that, on average, individuals from the general population scored “unsure,” “tends to be true,” or“ trends to be untrue” on DUREL items. Table 1 shows the demographic characteristics and scales of the participants.

All participants gave written informed consent. The Hungarian version of the scales, tests, and questionnaires was administered by trained experts who were naïve to the aim of the study (Perczel‐Forintos, Ajtay, Barna, Kiss, & Komlósi, 2018; Rózsa, Kő, Mészáros, Kuncz, & Mlinkó, 2010). The study was approved by the National Medical Research Council (Hungary) (ETT‐TUKEB 18814). Based on the permission of the National Medical Research Council, the study was also approved by the local ethics board.

### 2.2 Scales and questionnaires

#### 2.2.1 Mini International Neuropsychiatric Interview 7.0

We used the MINI 7.0, a brief structured clinical interview for 17 frequent mental disorders (Sheehan, 2015). The administration time is approximately 15–20 min. The MINI 7.0 is validated against the DSM‐5‐CV (Structured Clinical Interview for DSM‐5 Disorders—Clinician Version) in Hungarian (First, Williams, Karg, & Spitzer, 2016). Individuals with mental disorders were not included in the study.

#### 2.2.2 Duke University Religion Index

The Duke University Religion Index (DUREL) is a five‐item questionnaire for religious involvement as defined by the National Institute on Aging: organizational religious activity (ORA, 1 item), nonorganizational religious activity (NORA, 1 item), and intrinsic religiosity (IR, 3 items) (Koenig & Büssing, 2010). Participants rate the following questions: “(1) How often do you attend church or other religious meetings? (ORA) 1–Never; 2–Once a year or less; 3–A few times a year; 4–A few times a month; 5–Once a week; 6–More than once/week; (2) How often do you spend time in private religious activities, such as prayer, meditation or Bible study? (NORA) 1–Rarely or never; 2–A few times a month; 3–Once a week; 4–Two or more times/week; 5–Daily; 6–More than once a day; (3) In my life, I experience the presence of the Divine (i.e., God)–(IR) 1–Definitely not true; 2–Tends not to be true; 3–Unsure; 4–Tends to be true; 5–Definitely true of me; (4) My religious beliefs are what really lie behind my whole approach to life–(IR) 1–Definitely not true; 2–Tends not to be true; 3–Unsure; 4–Tends to be true; 5–Definitely true of me; (5) I try hard to carry my religion over into all other dealings in life (IR).” (Koenig & Büssing, 2010). The instrument has high test‐retest reliability (*r* = .90), and high internal consistence (Cronbach's alpha = 0.80–0.94).

#### 2.2.3 21‐item Peters et al. Delusion Inventory

The Peters et al. Delusion Inventory (PDI) consists of 21 items referring to different common delusional themes (Peters, Joseph, Day, & Garety, 2004). First, participants decide whether an item is true or not (e.g., “Do your thoughts ever feel alien to you in some way?”––yes or no; “Do you ever feel as if you are a robot or zombie without a will of your own?”––yes or no). If the item is true, they rate how distressing the belief or experience is, how often they think about it, and how true they believe it (min: 1, max: 5 points). The maximum total score is 315. The internal consistency (Cronbach's alpha = 0.85) and the test‐retest reliability (*r* = .82) of the scale are excellent. We used the total score as the independent measure.

#### 2.2.4 Beck Depression Inventory‐II

The Beck Depression Inventory‐II (BDI‐II) is a brief, self‐administered scale for depressive symptoms (administration time: 5 min) (Beck, Steer, & Brown, 1996). The scale consists of 21 items, each including four statements with increasing severity (e.g., “loss of interest: 0––I have not lost my interest in other people or activities; 1––I am less interested in other people or things than before; 2––I have lost most of my interest in other people or things; 3––It's hard to get interested in anything”). The total score is 63 (> 29––severe depression). The internal consistency (Cronbach's alpha = 0.82) and the test‐retest reliability (*r* = .80) of the scale are good. We used the total score as the independent measure.

#### 2.2.5 Wechsler Adult Intelligence Scale––III working memory index

The working memory index includes three tests: Digit Span, Letter‐Number Sequencing, and Arithmetic tests (Lange, 2011; Wechsler, 1997). The Digit Span test focuses on short‐term memory and attention (digits forward: participants repeat a series of numbers; digits reversed: participants repeat them in reverse order). In the Letter‐Number Sequencing test, the task is to repeat letters in alphabetical order and numbers in numerical order. Finally, in the Arithmetic test, participants perform mathematical operations to answer the questions (e.g., “If Jo has 12 buns, he then eats three and gives four away, how many does he have left?”).

### 2.3 Postdiction task

We used the Bear et al. (2017) procedure, which we modified to suit face‐to‐face administration. Stimuli were presented on a Display++ LCD monitor (Cambridge Research Systems) controlled by a Dell Precision T3640 workstation. The experiment ran in a Psychtoolbox3/MATLAB environment (MathWorks). Before the experiment, participants read a detailed explanation of the task described by Bear et al. (2017) and received 20 practice trials.

**Fig. 1:**
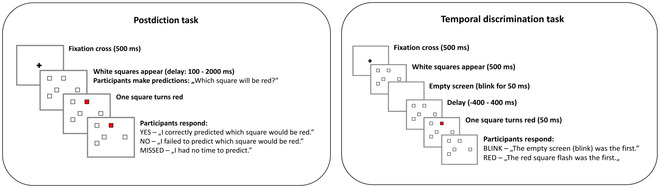
Postidiction and temporal discrimination tasks. In the postdiction task, following a brief fixation, five squares appeared on the screen in random locations. The task was to predict which of the squares would turn red after a delay phase. After the presentation of the red sqaure, participants responded whether they successfully predicted the square. In the temporal discrimination task, after fixation and presentation of squares, the screen blinked, or one of the squares turned into red (the figure illustrates the case when the blink was the first). The event that happened first (blinking or red square) was random. Finally, participants responded whether they observed the red square or the blink first.

An experimental trial comprised the following events (Fig. 1). First, a fixation cross (30‐pixel) was presented for 500 ms. Then, immediately after the fixation cross, five empty squares (50×50 pixels) appeared randomly on a 5×5 grid (20‐pixel space between each possible square location on the grid; the total display area: 330×330 pixels). The task was to “pick (in your head) a single square that you think will turn red” before one of the squares randomly turned red. There were four possible time intervals (delays) between the appearance of the five empty squares and the time point when one of these squares turned red: 100, 200, 400, and 2000 ms. We administered 20 trials at each delay period (80 trials altogether). The delay periods varied randomly across the 80 trials.

The response period began after a square turned red. First, we asked the observers to indicate whether they had correctly predicted the square that finally turned red. There were three response options: yes (participants pressed the key “i”), no (key “n”), and no time to make predictions (space bar). Then, participants moved to the subsequent trial by pressing the “enter” key after their response. The dependent variables were the probability of “yes” responses (probability of predicting the red square) and the probability of making any prediction by considering the ratio of missed trials (“no time”). The probability value ranged between 1 (the red square was successfully predicted in 100% of trials or there were predictions in 100% of trials––no missed trials) and 0 (the red square was successfully predicted in 0% of trials or there were predictions in 0% of trials––all trials missed).

### 2.4 Temporal discrimination task

This task served as a control for the prediction task to assess the temporal information processing abilities of the volunteers (Bear et al., 2017). As in the prediction task, a fixation cross (500 ms) preceded the presentation of the five empty squares. Following a 500‐ms presentation period of the squares, two possibilities happened randomly: the display became blank for 50 ms, or squares turned red for 50 ms. Following the blink or the red square, we inserted a delay period of 100, 200, or 400 ms, during which the empty squares appeared on the screen. The task was to indicate whether the blink (empty screen) was the first by pressing the key “v” or the red square was the first by pressing the key “p.” As in the postdiction task, there were 20 trials at each delay. The dependent variable was the probability of perceiving blink first at each delay in both conditions (blink first or red square first).

### 2.5 Data analysis

We used STATISTICA 13.1 (Tibco) for data analysis. Kolmogorov–Smirnov and Levene's tests were applied to investigate the normality of data distribution and the homogeneity of variance. For the comparison of the religious (intrinsic and nonintrinsic) and atheist groups, we conducted analyses of variance (ANOVAs) followed by *F*‐tests (planned comparisons) and Tukey's honestly significant difference (HSD) post hoc tests. In these analyses, the between‐subjects factor was the group (intrinsic, nonintrinsic, and atheist), and the within‐subjects factor was the delay. Separate ANOVAs were performed for the postdiction task and the temporal discrimination task. Pearson's product‐moment correlation coefficients were calculated between postdiction performances and demographic variables. In the general population, we investigated the predictors of intrinsic religiosity using multiple regression analysis (potential predictors: prediction task performances at short [100 ms] and long delays [2000 ms], PDI, BDI, BAI, age, gender, and education). The level of statistical significance was set at alpha < 0.05. In the graphs, we used 95% confidence intervals so as to give additional information to the null‐hypothesis testing statistics (Fidler & Loftus, 2009).

## 3 Results

### 3.1 Religious and atheist groups

First, we compared the postdiction effect as a function of delay between the presentation of squares and color change in the utmost intrinsically religious, less intrinsic religious, and atheist groups. There were significant main effects of group (*F*(2,147) = 19.0, *p* < .001, *η* ^2^ = 0.21) and delay (*F*(3,441) = 7.80, *p* < .001, *η* ^2^ = 0.05). The two‐way interaction between group and delay was not significant (*p* = .38).

**Fig. 2:**
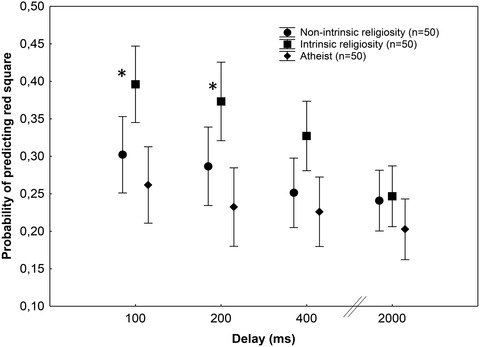
Mean probabilites of correctly predicting the red square at each delay in the postdiction task. Error bars indicate 95% confidence intervals. Individuals with high intrinsic religiosity outperformed the atheist group at 100 and 200 ms (*p < .05, Tukey's HSD tests).

Planned comparisons with *F*‐tests indicated significant differences between the intrinsic and nonintrinsic groups (*F*(1,147) = 14.48, *p* < .001), the intrinsic and atheist groups (*F*(1,147) = 37.22, *p* < .001), and the nonintrinsic and atheist groups (*F*(1,147) = 5.27, *p* < .05). Tukey's HSD tests yielded that the intrinsic religious group scored higher than the atheist group at 100 and 200 ms delays (*p* < .05). No other post‐hoc comparisons were significant (*p*s > .1) (Fig. 2). There were no significant correlations between postdiction performances and demographic measures (–.2 < *r*s < .2).

**Fig. 3:**
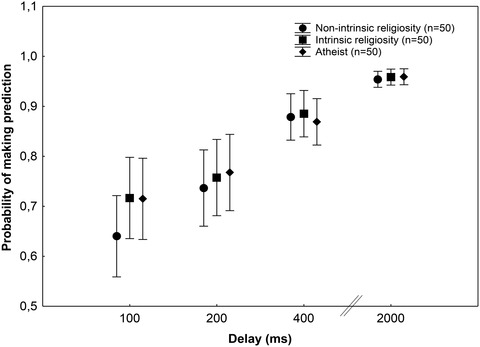
Mean probabilites of making predictions at each delay in the postdiction task. Error bars indicate 95% confidence intervals. There were no significant differences between the groups (*p* > .05).

**Fig. 4:**
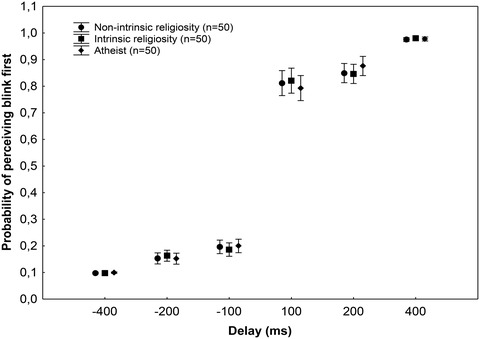
Results from the temporal discrimination task. The graph shows mean probabilities of perceiving the blink first at each delay. In positive delay values, the blink appeared first, whereas in negative delay values, the red square flashed first. There were no significant differences between the groups (*p* > .05).

We also analyzed two control measures: the probability of making predictions at each delay and temporal discrimination. The three groups showed similar probabilities to make predictions at each delay (*p* = .41) (Fig. 3). There were no significant between‐group differences in the temporal discrimination task (*p* = .96) (Fig. 4).

### 3.2 General population

Intrinsic religiosity was significantly predicted by postdiction at 100 ms delay (β* = 0.41, *SE* = 0.05, *r* = .41, *p*s < .001) and education (β* = 0.12, *SE* = 0.05; *p* < .05; *r* = .10, *p* = .06). The other potential predictors (postdiction at 2000 ms, PDI, BDI, BAI, age, and gender) were not significant. The whole model explained 17% of variance in intrinsic religiosity (*F*(9,340) = 9.07, *p* < .001, *R* ^2^ = .17).

To follow‐up the results of Bear et al. (2017), we investigated the association between postdiction and delusional thinking (PDI), and also added the measures of religiosity to the model. The PDI scores were predicted by postdiction task performance at 2000 ms delay (β* = 0.11, *SE* = 0.05; *p* < .05; *r* = .11, *p* < .05), but not by DUREL scores (organizational, private, and intrinsic religiosity, *p*s > .2). There were no significant correlations between DUREL scores, delusional thinking (PDI), depression (BDI), and anxiety (BAI) (–.2 < *r*s < .2).

## 4 Discussion

In a group of highly religious individuals and firm atheists, we found support for the central hypothesis: individuals with extreme intrinsic religiosity displayed enhanced postdiction at short temporal delays relative to atheists. Interestingly, the intrinsic religiosity–postdiction association was independent of delusions, and the religious and atheist groups achieved similar scores on the PDI scale measuring delusional thinking. To better understand this finding, it is reasonable to consider the nature of the scale items. In the PDI, there are only two items on which intrinsically religious people, by definition, score high (Peters et al., 2004): “Do you ever feel that you are especially close to God?” (item 8), and “Do you ever feel as if you have been chosen by God in some way?” (item 11). However, the other items of the PDI are not directly related to religiosity (e.g., “Do you ever feel as if people seem to drop hints about you or say things with a double meaning?,” or “Do you ever feel as if you are persecuted in some way?”), which explains why nonreligious people achieved comparable overall scores (e.g., someone may feel vigorously persecuted without any religious relevance).

Our findings are in concordance with the view that even extreme forms of religiousness are not necessarily associated with mental disorders (McCauley & Graham, 2020; Seitz, Angel, & Paloutzian, 2021): participants with high intrinsic religiosity reported that they entirely experienced the presence of God, their religious beliefs guided their whole existence, and they brought religion to every aspect of life, yet they were not more deluded than the atheists. In other words, enhanced postdiction was explicitly associated with intrinsic religiosity, but not delusions, in the highly religious sample. Therefore, mistiming of thought and perception may be a relevant low‐level factor in establishing mental representations related to the “sacred”. It is possible that forming nonempirical beliefs (e.g., religious representations) requires a blurred boundary between internal thoughts and external perceptions (Johnson, 1988). The postdiction task used in the present study taps on this mechanism because participants can easily confuse their internal thoughts (predictions about which square will be red) and external perceptions (the actual change of color), inducing a false impression that they correctly predicted color changes.

The question arises as to why in some individuals postdiction is tied to delusional thoughts, while in others, it is confined to intrinsic religiosity. The answer might come from cultural symbols interacting with cognitive representational systems, which are crucial in social‐cognitive development (Cerulo, Leschziner, & Shepherd, 2021). From our perspective, it is notable that our participants came from a highly religious milieu and regularly pursued their faith in congregations and churches. Historically embedded and societally accepted religious explanations nourished by culture and tradition may help interpret and cope with significant life events, unusual experiences, affects, and thoughts. This is a core aspect of the bright side of organizational religious activity (Geher & Wedberg, 2019). For example, individuals with high schizotypal traits (anomalous perceptual experiences, nonordinary beliefs, social introversion, extravagant and odd behavior, and emotional instability) may find a religious community framework and explanation to alleviate unpleasant feelings, thoughts, and maladaptive behavior (Hanel, Demmrich, & Wolfradt, 2019; Johnstone & Tiliopoulos, 2008; Mohr, Brandt, Borras, Gillieron, & Huguelet, 2006; Ng, 2007).

In a general population sample, we replicated the link between intrinsic religiosity and postdiction at short temporal delays. What do we know about the religious behavior of this group? According to the average scores on the DUREL scale (Koenig & Büssing, 2010), the volunteers from the general population reported that they were unsure regarding divine experience and the impact of religious beliefs on their life. Also, they attended religious gatherings a few times a year and performed prayer or spiritual meditation approximately once a week. In this sample, individuals with high education and pronounced postdiction tended to report that they were closer to the “Divine and sacred”. How is it related to delusive thinking in the population? When we combined religious measures and postdiction to assess the predictors of delusive thinking, scores on the delusion scale were predicted only by postdiction but, contrary to the expectation (Bear et al., 2017), at long temporal delays. Religiosity did not predict delusive thinking. Therefore, religious beliefs are linked to thought‐percept fusion when there is a short time between thought and perception, whereas delusive thinking is associated with thought‐percept fusion when the interval is long between thought and perception.

The present results suggest that the automatic (short temporal delay) phase of postdiction is linked to intrinsic religiosity, whereas the controlled (long temporal delay) phase is linked to delusive thinking. At short delays, estimating which square will be red is in progress when the actual physical change occurs (i.e., one of the squares turns red). Therefore, the ongoing predictive process interferes with perceptual changes resulting in the impression that the perceptual change was foreseen. In other words, there is an illusory reversal of internal prediction and predicted events in awareness. When the delay between estimation and physical change is long enough, the prediction process is complete, and the result is encoded in the working memory. In this case, the working memory content meets with the perceptual change, and the participant remembers the prediction when the square turns red (Bear et al., 2017). Our key finding raises the possibility that delusive thinking emerges when the working memory functions fail during prediction.

The postdiction––intrinsic religiosity association was independent of temporal discrimination, which resonates with the results of Bear et al. (2017), who found that delusion‐prone individuals did not perform worse on a temporal discrimination task than people with lower scores on the delusion scale. Analogously, individuals with outward intrinsic religiosity, less dominant intrinsic religiosity, and atheists displayed similar temporal discrimination performances.

A critical issue is how other models of belief formation are related to the theory of biased postdiction, a novel approach in both the CSR and the cognitive neuropsychiatry of delusions. Several belief formation theories stem from research on delusions, including attribution processes, inferential reasoning, belief evaluation, metacognition, error‐dependent updating, preconscious perceptual processing, and belief‐memory interferences (Connors & Halligan, 2014). For example, when inferential reasoning biases are present, people make premature conclusions on the truth‐value of events based on low levels of scarce evidence (“early jumping to conclusions”). On the other hand, individuals exhibiting firmly held beliefs often show weak metacognitive self‐monitoring and error‐driven updating of mental representations: they are less aware of incongruence between their beliefs and evidence from the real world and less likely to adapt their beliefs to reality. However, based on an accumulator model, Bear et al. (2017) argued that neither “early jumping to conclusions” nor ineffective prediction‐error processing and belief‐updating is sufficient to fully explain time‐dependent postdiction, a specific thought‐perception mistiming at short time intervals.

The role of perceptual postdiction in belief formation and delusive thinking resonates with Fleminger's (1992) model in which beliefs and expectations retune the interpretation of perceptual information. In the Fleminger model, expectations are not conscious, and individuals display a self‐reinforcing preconscious cycle of perceptual processing. In contrast, Stone and Young (1997) claimed that explicit beliefs alter the structure of perceptual experience (“we see what we believe”), and one may perceive the world as if beliefs were true without a chance of active inference and adaptive modification of representations. A similar phenomenon happens during postdiction: participants perceived the color change of the square as if it were consistent with their beliefs and expectations. However, in contrast to Fleminger's (1992) and Stone and Young's (1997) model, postdiction is based on percept‐thought mistiming and, consequently, a blurred boundary between internal representations and external reality Supporting Information.

In conclusion, we found new evidence that postdiction in visual awareness is associated with belief formation: it was associated with intrinsic religiosity in religiously committed people and the general population. Future studies are necessary to explore the relationship between postdiction and other formal models of belief formation under normal and pathological circumstances. It is especially relevant to gain a deeper insight into the relationship between thought‐percept fusion in early‐stage information processing, higher‐level attributions, narrative construction, and the social reinforcement of beliefs.

## Funding

The study received no external funding.

## Conflict of Interest

The author declares no conflict of interest.
